# Comprehensive genome-scale CRISPR knockout screening of CHO cells

**DOI:** 10.1038/s41597-025-04438-6

**Published:** 2025-01-15

**Authors:** Sung Wook Shin, Su Hyun Kim, Aghiles Gasselin, Gyun Min Lee, Jae Seong Lee

**Affiliations:** 1 Department of Molecular Science and Technology, Ajou University, Suwon, 16499 Republic of Korea; 2 Department of Biological Sciences, KAIST, Daejeon, 34141 Republic of Korea

**Keywords:** High-throughput screening, Functional genomics, Genomic analysis, Assay systems

## Abstract

Chinese hamster ovary (CHO) cells play a pivotal role in the production of recombinant therapeutics. In the present study, we conducted a genome-scale pooled CRISPR knockout (KO) screening using a virus-free, recombinase-mediated cassette exchange-based platform in CHO-K1 host and CHO-K1 derived recombinant cells. Genome-wide guide RNA (gRNA) amplicon sequencing data were generated from cell libraries, as well as short- and long-term KO libraries, and validated through phenotypic assessment and gRNA read count distribution. Additionally, we obtained gRNA amplicon sequencing data from the highly productive recombinant cell populations. By analyzing these datasets, essential genes involved in cell fitness as well as functional target genes associated with therapeutic protein production can be identified. Collectively, our next-generation sequencing datasets, derived from a robust and reliable CRISPR screening method, provide valuable insights into CHO genomic functions, advancing the development of next-generation CHO factories.

## Background & summary

The production of recombinant therapeutic proteins primarily relies on mammalian expression systems, with Chinese hamster ovary (CHO) cells being the main workhorse^1^. Advancements in automated clone screening and bioprocess optimization over the years have significantly improved the volumetric productivity and product quality of monoclonal antibodies^2^. Moreover, the advent of clustered regularly interspaced short palindromic repeats (CRISPR)/CRISPR-associated protein 9 (Cas9) technology, along with the recombinase-mediated cassette exchange (RMCE), has significantly advanced the development of recombinant CHO cell lines through site-specific integration (SSI)^3,4^.

Despite these technical strides, the rapidly growing biopharmaceutical market continues to demand novel and complex biomolecules. To enhance the production of difficult-to-express proteins, various genetic engineering strategies have been used to address cellular production bottlenecks, with targets identified through knowledge- and omics/prediction-based approaches^5,6^. However, these methods may not always yield optimal results because of potential biases or incomplete datasets. Moreover, a comprehensive understanding of the essential genomic regions in CHO cells remains elusive, limiting the development of a streamlined CHO factory with minimal genomes^7^. Therefore, an unbiased high-throughput genetic screening platform is essential for the development of next-generation CHO factories.

**Fig. 1:**
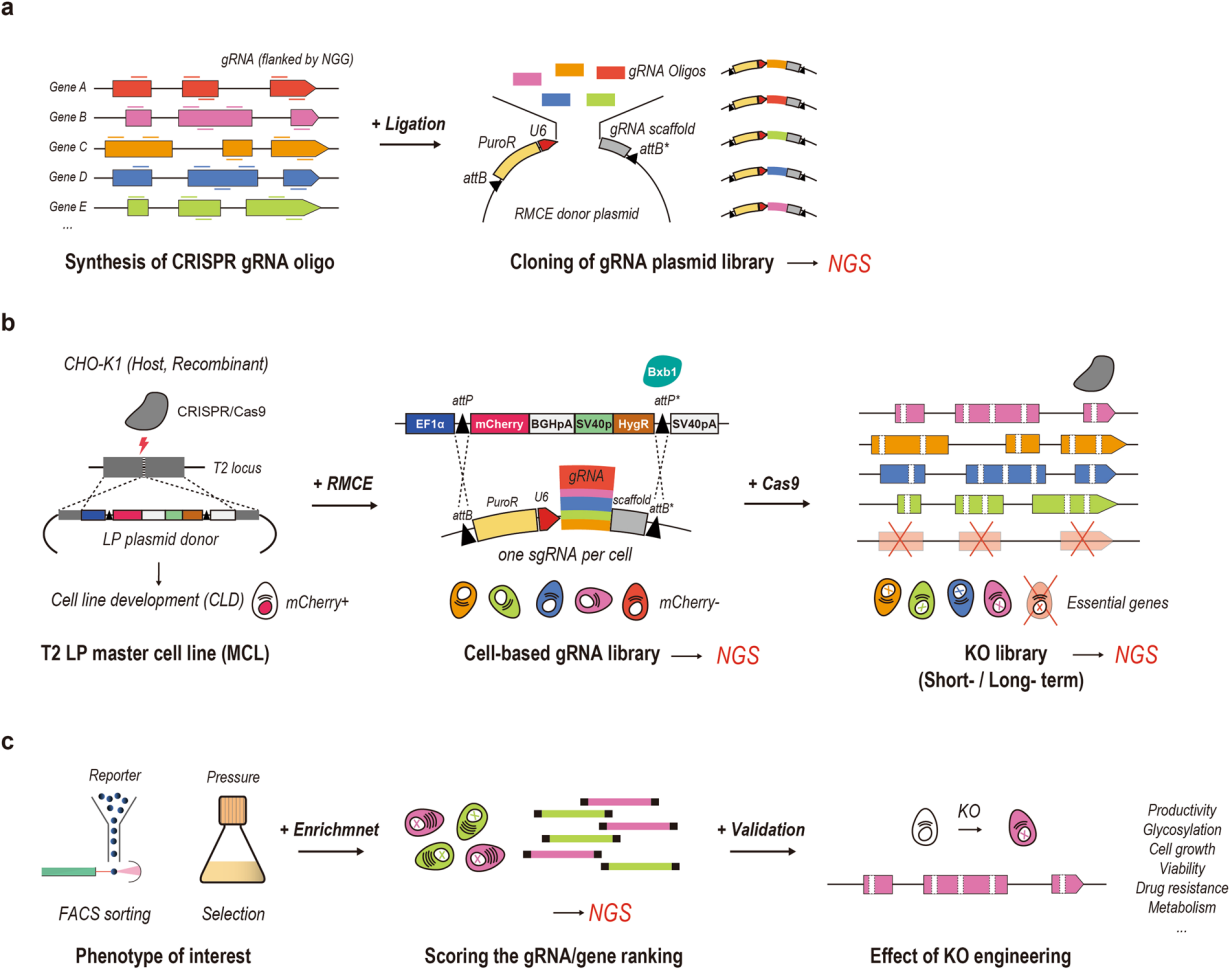
Schematic illustration of a non-viral, genome-scale CRISPR screening platform in Chinese hamster ovary (CHO) cells. (**a**) Plasmid library construction. CHO genome-wide CRISPR guide RNA (gRNA) oligonucleotides were designed and synthesized. Recombinase mediated cassette exchange (RMCE) donor plasmid library was then constructed by cloning gRNA oligos into a backbone plasmid that contained a promoter-less puromycin resistance (PuroR) gene and gRNA scaffold, flanked by attB and mutant attB recombination sites. The plasmid library was verified using next-generation sequencing (NGS) analysis. (**b**) Workflow of cell library and knockout (KO) library development. The CHO-K1 host and recombinant cell-based master cell lines (MCLs) were established through CRISPR/Cas9-mediated site-specific integration (SSI) of the landing pad (LP) plasmid donor, which contained mCherry and hygromycin resistance (HygR) gene flanked by attP and mutant attP recombination sites. The host and recombinant cell libraries were generated through the Bxb1-att recombination system and puromycin selection. Promoter-trapping ensures the expression of a single gRNA per cell. The host and recombinant KO libraries were then generated through transient Cas9 expression and blasticidin semi-selection. The distribution of gRNAs in the pooled cell libraries and KO libraries was confirmed using NGS analysis. The KO libraries were also analyzed after long-term cultivation. (**c**) Functional genomic screens. Phenotypes of interest in the KO library can be enriched through reporter-based fluorescence-activated cell sorting (FACS) or pool selection under selective pressure. Highly productive recombinant cell populations were sorted in this study. The distribution of gRNAs was confirmed using NGS analysis. The screening hits are candidate targets for KO cell engineering.

Pooled CRISPR screening has emerged as a powerful tool in functional genomics. This forward genetic approach involves introducing genetic perturbations into cells and analyzing them using next-generation sequencing (NGS) before and after phenotypic selection. Previously, we developed a virus-free, RMCE-based CRISPR knockout (KO) screening method (Fig. 1)^8^. This platform utilizes RMCE to introduce a guide RNA (gRNA) library into a master cell line (MCL), generating a cell library. After transient Cas9 expression, the cell library undergoes KO to generate a KO library.

**Fig. 2:**
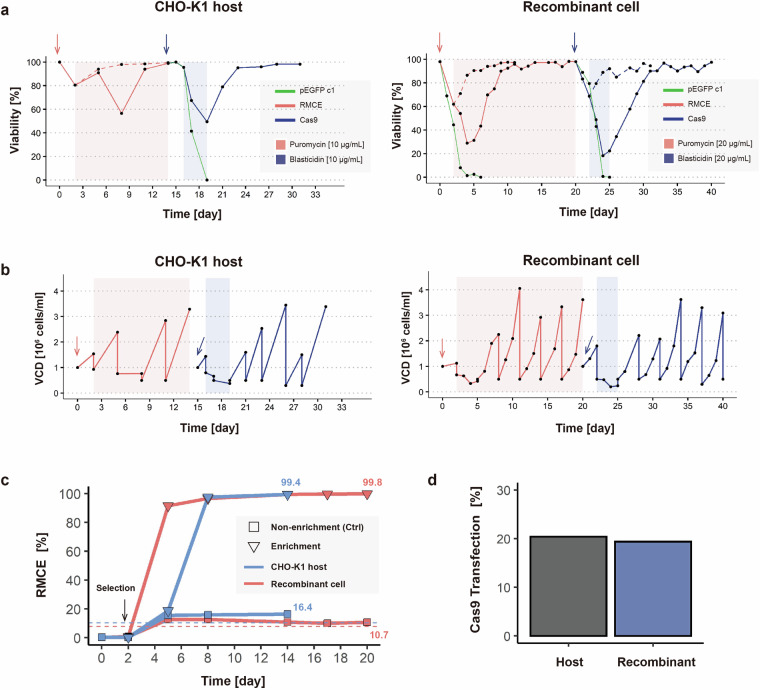
The development process of the cell and KO libraries using a non-viral genome-scale CRISPR screening platform. Entire profiles of (**a**) viability and (**b**) cell growth (VCD; viable cell density) during the generation of the cell (red solid lines) and KO (blue solid lines) libraries in both CHO-K1 host and recombinant cells. Arrows indicate the transfection of RMCE (red) and Cas9 (blue). Shaded areas denote periods of chemical treatment for cell library enrichment (red, puromycin) and KO library enrichment (blue, blasticidin). The pEGFP-c1 expression vector was used as the transfection control (green lines), while chemically untreated cells served as non-enriched controls (dotted lines). (**c**) The percentage of mCherry-negative populations in the CHO-K1 host (blue line) and recombinant cells (red line) during cell library development with and without puromycin selection. Dotted line indicates the threshold required to maintain 500 × gRNA coverage. The mCherry negativity in enriched populations indicated successful implementation of the genome-scale plasmid library into cells. (**d**) Bar plot showing the transfection efficiency of Cas9 in CHO-K1 host cells and recombinant cells as measured through 2 A peptide-linked reporter expression.

**Fig. 3:**
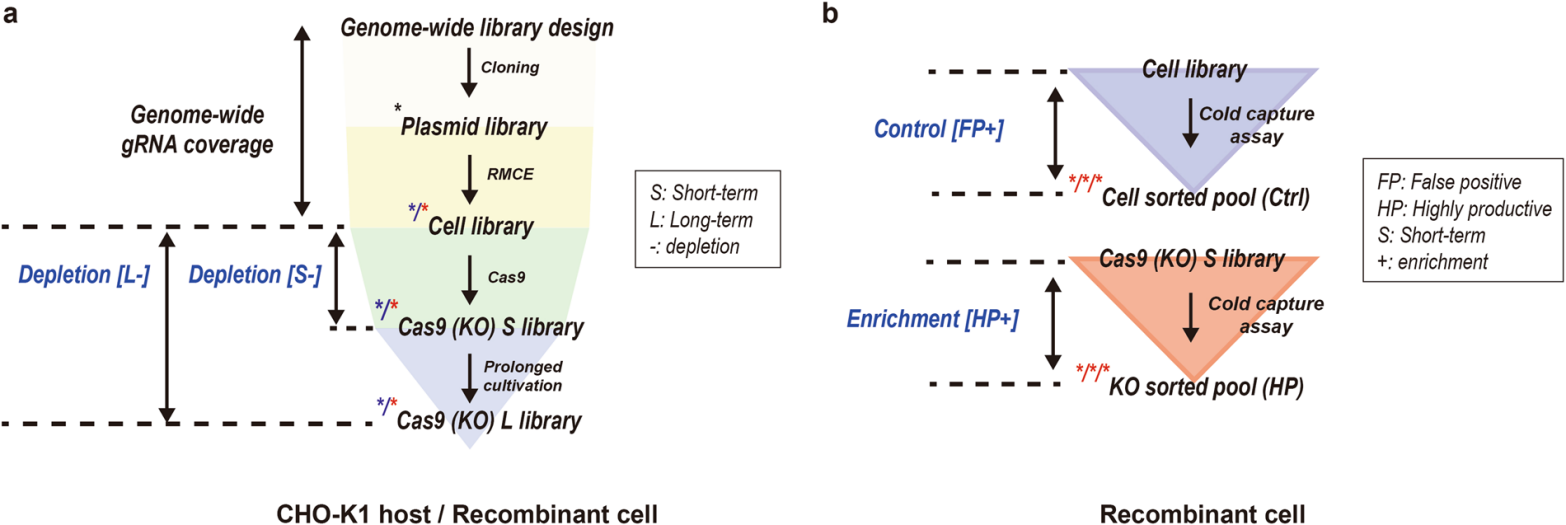
Overview of gRNA library NGS data collection. (**a**) Data collection during cell and KO library generation. The CHO genome-wide gRNA library, containing 111,651 unique gRNAs targeting 21,585 genes, was designed, cloned, and introduced into CHO-K1 host and recombinant cells via RMCE. Cell libraries underwent genome-wide KO (Cas9 short-term library) and prolonged cultivation (Cas9 long-term library) for data collection. Genome-wide gRNA representation in plasmid and cell libraries was validated, and gRNA distribution in the Cas9 short-term and long-term libraries was analyzed. Core and conditional essential genes can be identified as significantly depleted genes, depending on the specific cell lines and culture durations. (**b**) Data collection during positive selection. The highly productive recombinant Cas9 short-term library was enriched using the cold capture secretion assay. A control recombinant cell library underwent the same experimental procedure to exclude false positive hits. Functional genes associated with the phenotype of interest were identified as significantly enriched gRNAs. The generated NGS dataset is indicated by asterisks, with the number of asterisks representing the biological replicates: black for the plasmid library, blue for CHO-K1 host, and red for recombinant cells. In total, 13 NGS datasets were collected. S, short-term; L, long-term; FP, false positive; HP, highly productive; -, significantly depleted gRNAs; and + , significantly enriched gRNAs.

**Fig. 4:**
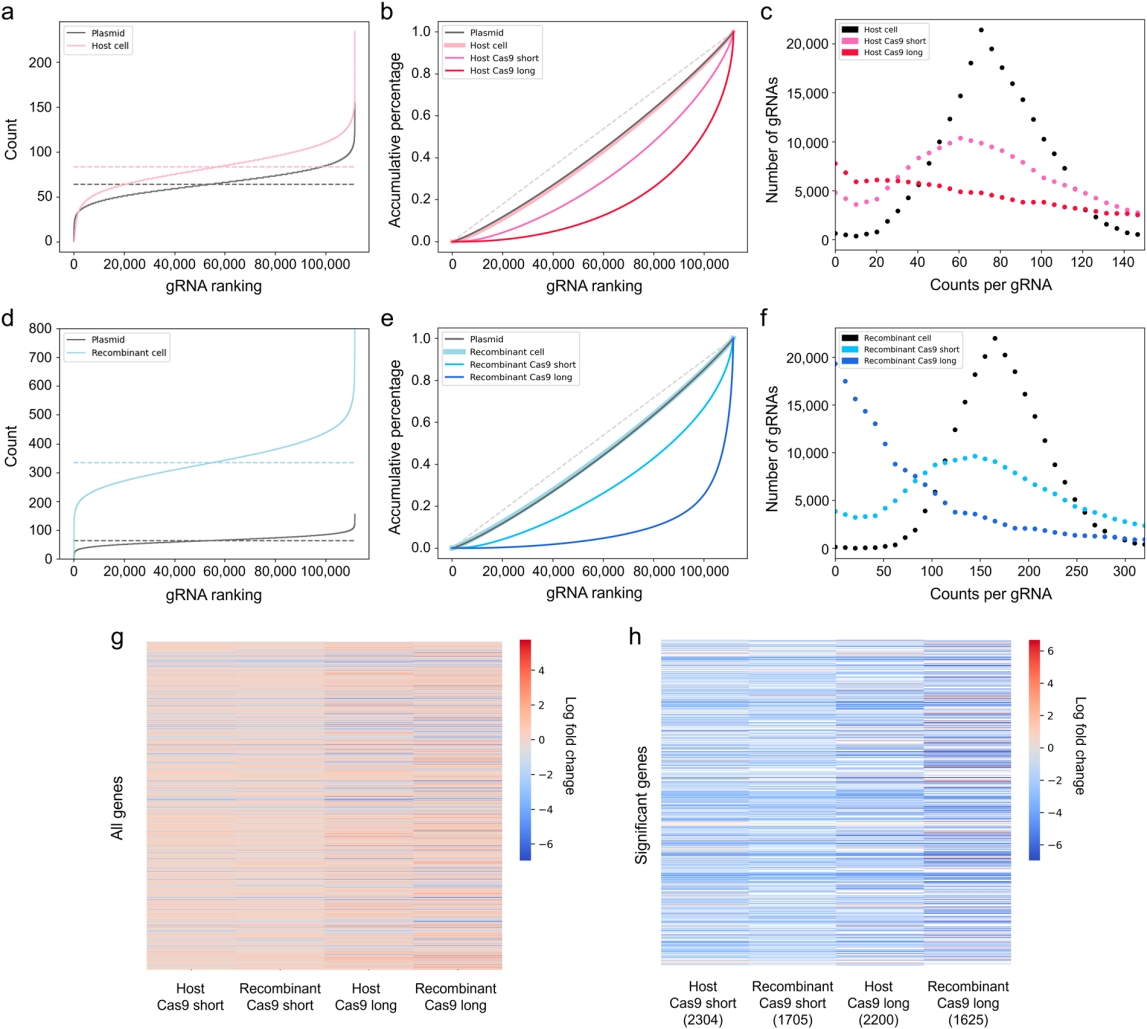
Representation of read count distribution. CHO-K1 host and recombinant cells were transfected with the plasmid library and Bxb1 recombinase to generate a cell library. The cell library pool was transfected with a Cas9 plasmid. After 16 d, Cas9 short-term library cells were generated. After 37 d, a Cas9 long-term library cells were generated. The gRNA sequences in each library cell pool were sequenced using NGS. (**a**) Read count number of each gRNA within the plasmid and host cell libraries. (**b**) Cumulative percentages of sequencing reads in the plasmid, host cell, host Cas9 short-term, and host Cas9 long-term libraries. (**c**) Read count distributions of gRNAs after Cas9 transfection in the host cell, host Cas9 short-term, and host Cas9 long-term libraries. (**d**) The read count number of each gRNA within the plasmid and recombinant cell libraries. (**e**) Cumulative percentages of sequencing reads in the plasmid, recombinant cell, recombinant Cas9 short-term, and recombinant Cas9 long-term libraries. (**f**) Read count distributions of gRNAs after Cas9 transfection in the recombinant cell, recombinant Cas9 short-term, and recombinant Cas9 long-term libraries. In a, b, d, and e, the dashed lines indicate an ideal distribution in the gRNA library. Heat maps showing fold changes (FC) in (**g**) all genes and (**h**) statistically significant genes from MAGeCK α-RRA analysis. The gRNA read counts from the host Cas9 short-term and Cas9 long-term libraries were compared to those from the host cell library. The gRNA read counts from the recombinant Cas9 short-term and Cas9 long-term libraries were compared to those from the recombinant cell library. A gene was considered significant at a *P*-value threshold of 0.01. FC are represented on a color gradient scale from red to blue. The N/A value was assigned a white color. Cell, cell library after plasmid library transfection; Cas9 short, Cas9 short-term library after 16 d; and Cas9 long, Cas9 long-term library after 37 d.

**Fig. 5:**
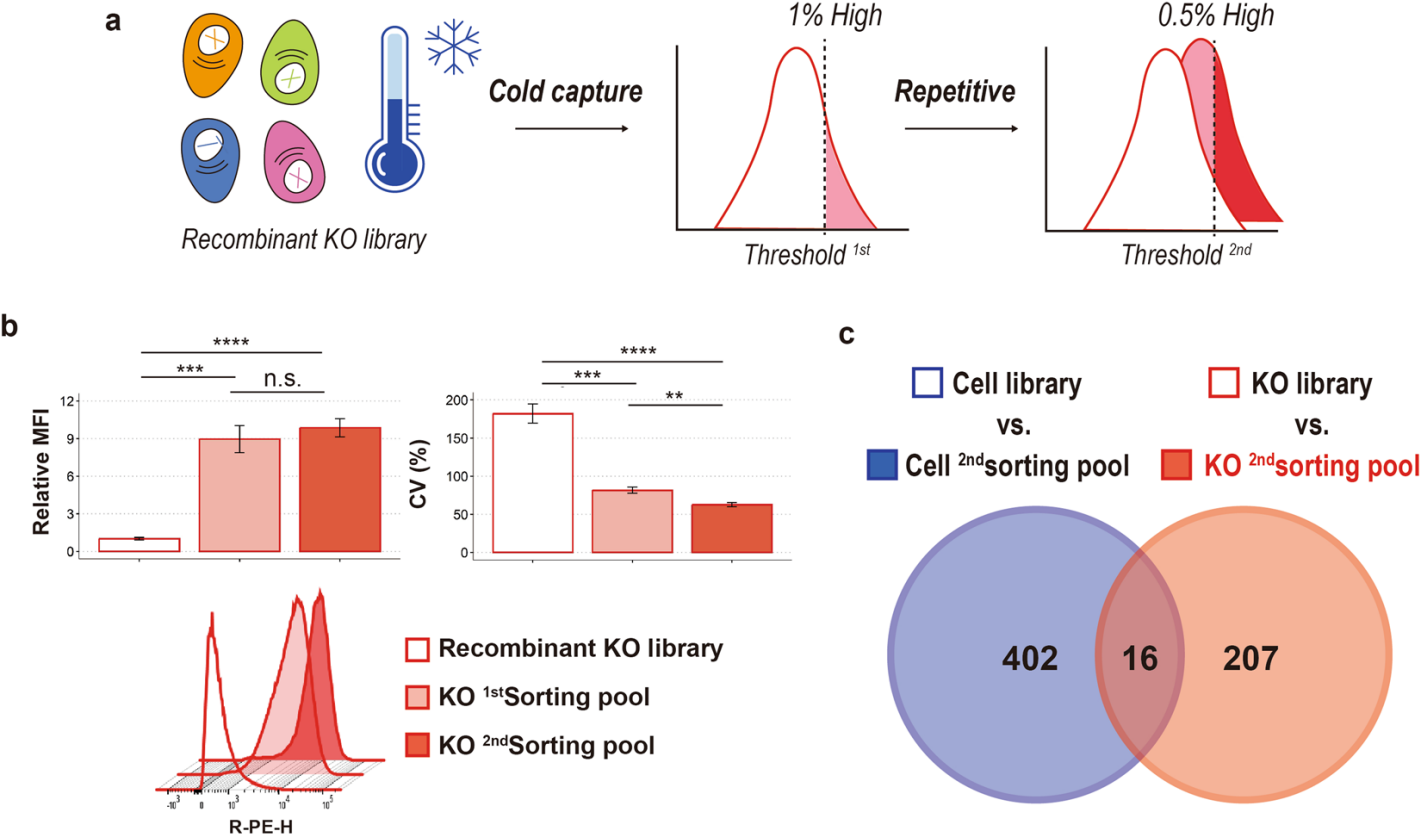
Enrichment of highly productive (HP) cell populations. (**a**) Experimental scheme for HP population enrichment. Two rounds of HP population enrichment were performed using a cold capture secretion assay-based FACS method. The gating threshold targeted the top 1% and 0.5% of the KO library for the first and second rounds of sorting, respectively. A similar procedure was applied to the cell library as a control. (**b**) The relative mean fluorescent intensity (MFI) and coefficients of variation (CV) values during the cold capture assay for HP enrichment. R-phycoerythrin (R-PE) expression in the recombinant KO library, as well as the 1^st^ and 2^nd^ HP KO sorting pools, was analyzed. Error bars represent the standard deviations (SD) from three independent experiments. Representative histograms of the KO library and KO sorting pools are also shown. Statistical significance of the mean differences was determined using an unpaired two-tailed t-test, n.s. *P* > 0.05, **P < 0.01, ***P < 0.001, and ****P < 0.0001. (**c**) Venn diagram representing the number of significantly enriched genes in the HP sorting pools (n = 3). The 2^nd^ HP sorting pool of the KO library was compared against the parental KO library, while the 2^nd^ HP sorting pool of the control cell library was compared against the parental cell library.

In this study, we performed an RMCE-based pooled CRISPR KO screen using a genome-wide gRNA library, in both CHO-K1 host and CHO-K1 derived recombinant cells. Following optimized experimental procedures for cell library and KO library development (Fig. 2), we generated genome-wide gRNA amplicon sequencing data. Specifically, NGS datasets were obtained from short-term (day 16) and long-term (day 37) KO libraries in both CHO-K1 host and recombinant cells (Fig. 3a). To ensure comprehensive representation of each gRNA within the pooled libraries, technical validation was conducted through phenotypic assessment (Fig. 2c) and read count distribution analysis (Fig. 4). Furthermore, we performed positive selection screening using recombinant cells to enrich highly productive (HP) cell populations (Fig. 5), thereby generating additional NGS datasets for functional genomic analysis (Fig. 3b).

Consequently, we generated 13 NGS datasets, as summarized in Fig. 3. These include the plasmid library, cell library (host and recombinant), short-term KO libraries (host and recombinant), long-term KO libraries (host and recombinant), and HP populations in triplicates (recombinant cell library and recombinant KO library). The gRNA amplicon sequencing datasets allow for the identification of core and conditional essential genes^9,10^, as well as functional target genes associated with specific phenotypes of interest^8^, by analyzing significantly depleted or enriched gRNAs or genes across samples (Fig. 3).

## Methods

### CHO genome-wide CRISPR KO gRNA library construction

The CHO genome-wide CRISPR KO gRNA library contained 111,651 unique gRNAs targeting 21,585 genes. The library was designed as previously described (Fig. 1a)^11^. The designed library was synthesized as an oligo pool by Twist Bioscience (San Francisco, CA, USA). The gRNA oligo pool was amplified using Phusion High-Fidelity polymerase chain reaction (PCR) Master Mix (Thermo Fisher Scientific, Waltham, MA, USA) (98 °C for 30 s; 20 × cycles, 98 °C for 10 s, 63 °C for 30 s, 72 °C for 1 min; and 72 °C for 3 min), and purified from 2% agarose gel using NucleoSpin Gel and PCR Clean-up (Macherey-Nagel, Duren, Germany). The purified gRNA oligo pool was cloned into a plasmid backbone, which was digested with FastDigest Esp3I enzyme (Thermo Fisher Scientific), using Gibson Assembly Master Mix (New England Biolabs, Ipswich, MA, USA). The cloned plasmid pool was purified and concentrated using the isopropanol precipitation method as previously described (Fig. 1a)^12^. To amplify the pooled gRNA library, the purified plasmid pool was electroporated into Endura ElectroCompetent Cells (Lucigen, Middleton, WI, USA) using a MicroPulser Electroporator (Bio-Rad, Hercules, CA, USA). The resulting cells were cultured in Yeast Extract Tryptone medium overnight at 37 °C. Electroporation efficiency was calculated as previously described^12^, and the transformation step was repeated to obtain >500 colonies per gRNA in the library. Plasmid DNA was purified using a NucleoBond Xtra Maxi EF Kit (Macherey-Nagel), according to the manufacturer’s instructions.

### Cell lines, culture maintenance, and culture media

CHO-K1 host cells were cultured in Dulbecco’s modified Eagle’s medium (DMEM; Gibco, Grand Island, NY, USA) supplemented with 7% fetal bovine serum (FBS; HyClone, Logan, UT, USA). The CHO-K1 derived recombinant cell line producing EPO-fc was created as previously described^13^. Briefly, the dihydrofolate reductase (DHFR) KO CHO-K1 cell line was subjected to CRISPR/Cas9-mediated SSI of a DHFR-EGFP landing pad (LP) construct at the *C12orf35* locus. Subsequently, Cre recombinase-mediated RMCE of CMV-EGFP into CMV-EPO-fc cells yielded a recombinant cell line. Recombinant cells were cultured in DMEM supplemented with 10% dialyzed FBS (dFBS; Gibco, Grand Island, NZ origin). The CHO-K1 host and recombinant cells were maintained in T25 flasks at 37 °C in a humidified 5% CO_2_ atmosphere.

After the CRISPR/Cas9-mediated SSI of the mCherry LP construct, the generated MCLs were grown under serum-free suspension culture conditions. The serum-free adapted host MCL was cultured in CD-CHO medium (Gibco) supplemented with 4 mM glutamine (HyClone) and 100 × anti-clumping agent (Lonza, Basel, Switzerland). Serum-free adapted recombinant MCL were cultured in PowerCHO2CD medium (Lonza) supplemented with 8 mM glutamine (HyClone). The MCLs (host, recombinant) were cultured in 125 mL Erlenmeyer flasks (Corning, Corning, NY, USA) at 110 rpm, 37 °C in a humidified 5% CO_2_ atmosphere. Viable cell density (VCD) and viability were measured using a Countess II FL automated cell counter (Invitrogen, Carlsbad, CA, USA) with the trypan blue dye exclusion method.

### Generation of the T2 site LP MCLs

The MCLs were generated by CRISPR/Cas9-mediated SSI of the mCherry LP construct at the T2 site (Fig. 1b, left)^14^. Before transfection, the CHO-K1 host (0.5 × 10^6^ cells/mL) was seeded in T25 flasks. After 24 h, the cells were transfected with an mCherry LP donor plasmid, a gRNA plasmid targeting the noncoding region (T2 site), and a Cas9 plasmid at a ratio of 1:1:1 (w/w) using Lipofectamine 2000 (Invitrogen). Recombinant cells (0.2 × 10^6^ cells/mL) were seeded in six-well plates. After 24 h, the cells were transfected with the mCherry LP donor plasmid and gRNA/Cas9 expression vector targeting the T2 site at a ratio of 1:1 (w/w) using Lipofectamine 3000 (Invitrogen). All transfections were performed according to the manufacturer’s instructions. After 48 h, the transfected CHO-K1 host (0.1 × 10^6^ cells/mL) and recombinant cell (0.15 × 10^6^ cells/mL) pools were seeded in T25 flasks with an addition of 800 and 600 µg/mL hygromycin (Clontech, San Jose, CA, USA), respectively. During the 2 weeks of selection, the selective medium was changed every 3 d. After recovery, the host MCL pool was sorted based on mCherry positive/ZsGreen negative expression using fluorescence-activated cell sorting (FACS)Aria II (BD Biosciences, San Jose, CA, USA), followed by limiting dilution (0.3 cells/well), whereas the recombinant MCL pool was directly subjected to limiting dilution (0.4 cells/well) in a 96-well plate. Clones were expanded and validated based on several criteria, including the results of 5′/3′-junction PCR, Sanger sequencing, single copy integration, and flow cytometry analyses. The final MCLs (host and recombinant) were grown in serum-free suspension cultures.

### Generation of cell-based gRNA libraries

Cell-based gRNA libraries were generated by introducing the gRNA library into MCLs (Fig. 1b, middle). For the gRNA library RMCE in MCLs (host, recombinant), cells were seeded at a density of 1.0 × 10^6^ cells/mL in 125 mL Erlenmeyer flasks, each containing 50 mL SFM4Transfx-293 medium (HyClone) supplemented with 4 or 8 mM glutamine on the day of transfection. To guarantee coverage of 500 cells per gRNA, the number of cells required for transfection was determined based on the measured RMCE efficiency (Fig. 2c). A total of 3.0 × 10^8^ host and 7.5 × 10^8^ recombinant MCLs were transfected with the gRNA plasmid library and the NLS-Bxb1 recombinase plasmid at a ratio of 3:1 (w/w) using 293fectin (Thermo Fisher Scientific), according to the manufacturer’s instructions. After 48 h, the transfected MCLs (host, recombinant) were treated with 10 and 20 μg/mL puromycin (Sigma-Aldrich, St. Louis, MO, USA) for the enrichment of cell-based gRNA libraries. During selection, cell pools were maintained at a density of 0.5 × 10^6^ cells/mL every 3 d (Fig. 2a,b), and the percentage of mCherry-negative population was assessed using LSRFortessa (BD Biosciences, San Jose, CA, USA) (Fig. 2c). After 12 and 18 d of selection, respectively, the genomic DNA of the host and recombinant cell-based gRNA libraries were harvested for NGS analysis (Fig. 1b, middle).

### Generation of cell-based KO libraries

KO libraries were generated by Cas9 expression in cell-based gRNA libraries (Fig. 1b, right). For Cas9 transfection in cell-based libraries (host and recombinant), cells were seeded at a density of 1.0 × 10^6^ cells/mL in 125 mL Erlenmeyer flasks, each containing 50 mL of SFM4Transfx-293 medium (HyClone) supplemented with 4 or 8 mM glutamine on the day of transfection. To guarantee coverage of 500 cells per gRNA, the number of cells required for transfection was determined based on the measured Cas9 transfection efficiency (Fig. 2d). A total of 3.0 × 10^8^ host and recombinant cell-based libraries were transfected with the EGFP-T2A-Cas9/blasticidin (BSD) plasmid, using 293fectin, according to the manufacturer’s instructions. After 24- or 48-h post-transfection, the transfected cell libraries (host, recombinant) were treated with 10 and 20 μg/mL blasticidin (Sigma-Aldrich), respectively, for the enrichment of KO libraries. After 3 d, the cells were recovered in a fresh medium. During recovery, cell pools were maintained at a density of 0.3–0.5 × 10^6^ cells/mL every 3 d (Fig. 2a,b). Transient Cas9 expression was monitored based on 2 A peptide-linked reporter expression using an LSRFortessa (BD Biosciences) (Fig. 2d). The host KO library was subjected to genomic DNA extraction for NGS analysis 16 (host Cas9 short-term) and 37 (host Cas9 long-term) d after Cas9 expression (Fig. 1b, right). Genomic DNA was extracted from the recombinant KO library for NGS analysis after 17 (recombinant Cas9 short-term) and 37 (recombinant Cas9 long-term) d after Cas9 expression (Fig. 1b, right).

### Enrichment of HP recombinant cell population

To enrich the HP cell populations from the recombinant KO library, two rounds of FACS sorting were conducted using a cold capture secretion assay (Fig. 5a,b)^15^. In the first round, 9.6 × 10^7^ cells were harvested to guarantee 500× representation of gRNAs and centrifuged at 500 rcf for 3 min at 4 °C. The cell pellets were washed with cold PBS, followed by washing with cold PBS containing 1% polyvinylpyrrolidone (PVP-40; Sigma-Aldrich). Cells were then incubated with cold PBS + 1% PVP-40 + 1% R-phycoerythrin (R-PE) conjugated Fcγ specific anti-human IgG F(ab’)_2_ fragment antibody (Jackson ImmunoResearch Laboratories, WestGrove, PA, USA) at 100 μL per 1.0 × 10^6^ cells, for 15 min in the dark on ice. Stained cells were washed twice with cold PBS containing 1% PVP-40, and resuspended in 2.5 mL of a 1:1 (v/v) mixture of PBS and culture medium supplemented with 1% dFBS and 1% antibiotic-antimycotic solution (Gibco). The top 1% of the HP cell populations (high R-PE) were bulk-sorted using FACSAria Fusion (BD Biosciences). After recovery, the second round of FACS sorting was conducted with 8.0 × 10^7^ 1^st^ HP sorting pools, setting a threshold to isolate the top 0.5% HP cell populations from the recombinant KO library. After recovery, 4 × 10^6^ cells from 2^nd^ HP sorting pool were subjected to genomic DNA extraction for NGS analysis (Fig. 1c, middle). The recombinant cell library was subjected to the same experimental procedure as a control. All experiments were performed in triplicate.

### Preparation of NGS samples

Genomic DNA was extracted using the Exgene Blood SV kit (GeneAll Biotechnology, Seoul, Korea), following the manufacturer’s instructions. To prepare NGS samples, PCR was performed in a total volume of 50 μL with 3–4 μg genomic DNA per reaction using NEBNext Ultra II Q5 Master Mix (New England Biolabs) (98 °C for 3 min; 22 cycles, 98 °C for 10 s, 60 °C for 30 s, 72 °C for 30 s; 72 °C for 5 min), as previously described^11,16^. PCR products were purified using a NucleoSpin Gel and PCR Purification Kit (Macherey-Nagel) and indexed using a TruSeq Nano DNA Library Prep Kit (Illumina, San Diego, CA, USA). The indexed library was quantified using qPCR following the Illumina qPCR Quantification Protocol Guide (Illumina). The library size was determined using a TapeStation D1000 ScreenTape (Agilent Technologies, Santa Clara, CA, USA) and sequenced on a HiSeq X Ten sequencer (Illumina).

### NGS data analysis

For the computational analysis of gRNA libraries, raw FASTQ files were analyzed using a Model-based Analysis of Genome-wide CRISPR/Cas9 Knockout (MAGeCK)^17^. MAGeCK v0.5.9.5, was run according to the instructions (https://sourceforge.net/p/mageck/wiki/Home/#usage). For comparative analysis, the gRNA read counts from the Cas9 short-term and Cas9 long-term libraries were compared with those from the cell library using the MAGeCK α-RRA algorithm. A gene was considered significant at a *P*-value threshold of 0.01. For functional genomic screening analysis, NGS data from the sorting pools were analyzed using PinAPL-Py (http://pinapl-py.ucsd.edu/)^18^. All parameters were set to default values.

## Data Records

The raw gRNA FASTQ files have been deposited in the Sequence Read Archive (SRA) database of the National Center for Biotechnology Information (NCBI) under the accession number SRP509673^19^. The raw gRNA FASTQ file of plasmid library has been deposited under the accession number SRP446953^20^. A list of accession number is summarized in Table 1.

**Table 1:** A list of NCBI accession number.

Library	Accession	Study	Sample name	Biosample accession
plasmid	SRR25109622	SRP446953	SAMN36265897	Plasmid library
host_cell	SRR29155703	SRP509673	SAMN41517651	Host cell library
host_cas9_short	SRR29155702	SRP509673	SAMN41517652	Host Cas9 short term
host_cas9_long	SRR29155699	SRP509673	SAMN41517653	Host Cas9 long term
recombinant_cell	SRR29155698	SRP509673	SAMN41517654	Recombinant cell library
recombinant _cas9_short	SRR29155697	SRP509673	SAMN41517655	Recombinant Cas9 short term
recombinant _cas9_long	SRR29155696	SRP509673	SAMN41517656	Recombinant Cas9 long term
2^nd^_cell_sorting_pool_1	SRR29155695	SRP509673	SAMN41517657	Recombinant KO sorted replicate 1
2^nd^_cell_sorting_pool_2	SRR29155694	SRP509673	SAMN41517658	Recombinant KO sorted replicate 2
2^nd^_cell_sorting_pool_3	SRR29155693	SRP509673	SAMN41517659	Recombinant KO sorted replicate 3
2^nd^_KO_sorting_pool_1	SRR29155692	SRP509673	SAMN41517660	Recombinant cell sorted replicate 1
2^nd^_KO_sorting_pool_2	SRR29155701	SRP509673	SAMN41517661	Recombinant cell sorted replicate 2
2^nd^_KO_sorting_pool_3	SRR29155700	SRP509673	SAMN41517662	Recombinant cell sorted replicate 3

## Technical Validation

### Amplicon sequencing read quality validation and assessment of gRNA library representation

To assess the representation of the gRNA library, gRNA sequences in library plasmids or genomic DNA of cells were extracted, amplified, and sequenced using NGS. FastQC was used for the quality assessment of raw reads. The total number of raw reads was 9.9 million to 57.9 million per library (Table 2). PCR amplicon samples from the gRNA library had an average read length of 151 bp. The MAGeCK was utilized for read trimming, read alignment, counting, and normalization. The total number of mapped reads was 3.6–24.8 million per library after mapping, with a mapping percentage ranging 36.5–45.5%. To assess the gRNA library distribution generated using the RMCE-based platform, the abundances of gRNAs were quantified. The cell-based gRNA library in host and recombinant cell lines showed an even distribution similar to that of the plasmid library with a coverage of 99.7% and 99.9% and a skew ratio of 1.98 and 1.78, respectively, indicating sufficient gRNA library distribution (Fig. 4a,d). After Cas9 transfection, the gRNA library distributions of the Cas9 short-term and Cas9 long-term libraries were disrupted, showing a gradual skew and higher Gini index (Fig. 4b,c,e,f). To ensure the statistical significance of the gRNA distribution results at the gene level, comparative analyses were conducted. The gRNA profiles of the Cas9 short-term and Cas9 long-term libraries were compared with those of the cell library. Genes exhibited more pronounced FC in long-term cultures compared to short-term cultures across both host and recombinant cell lines (Fig. 4g). When filtering genes with statistical significance (*P* < 0.01), the degree of FC was greater in the long-term cultures than in short-term cultures (Fig. 4h). Therefore, the dataset generated from the host and recombinant cell libraries demonstrates significantly different gRNA distributions.

**Table 2:** Statistics of read counts in CHO-K1 host and recombinant libraries.

Library	Reads	Mapped	Percentage	Gini index	Sequence length
plasmid	9,924,103	3,625,419	0.3653	0.04591	35–246
host_cell	10,599,311	4,756,165	0.4487	0.04953	151
host_cas9_short	48,887,560	19,002,580	0.3887	0.09136	151
host_cas9_long	42,547,961	15,518,319	0.3647	0.1884	151
recombinant_cell	48,880,530	21,184,631	0.4334	0.02756	151
recombinant _cas9_short	57,894,047	24,838,400	0.429	0.09101	151
recombinant _cas9_long	41,528,731	18,894,263	0.455	0.2574	151

### Assessment of functional gene enrichment using FACS

To obtain a dataset for functional genomic screening, we performed cold capture assay on a recombinant KO library (Fig. 5a,b). After two rounds of FACS sorting, the gRNA sequences from sorting pools were analyzed using NGS. A control recombinant cell library was subjected to the same positive selection procedure, where the population was presumed to remain unaffected by functional gene KO. The total number of raw read counts ranged from 4.9 to 5.8 million, with 2.3 to 2.7 million mapped read counts (Table 3). To ensure the reliability of the experimental results (i.e. functional gene enrichment), we compared the number of significantly enriched genes between the cell and KO 2^nd^ sorting pools. Minimal overlap was observed between the significantly enriched targets in the control cell sorting pool and those in the KO sorting pool (Fig. 5c), which indicates that the significantly enriched genes unique to the KO sorting pools represent candidate targets for HP CHO cell engineering.

**Table 3:** Statistics of read counts in 2^nd^ cell and KO sorting pools.

Library	Reads	Mapped	Percentage	Gini index	Sequence length
2^nd^_cell_sorting_pool_1	5,618,642	2,655,809	0.4727	0.9715	151
2^nd^_cell_sorting_pool_2	4,855,207	2,390,047	0.4923	0.9808	151
2^nd^_cell_sorting_pool_3	5,216,077	2,316,071	0.444	0.9534	151
2^nd^_KO_sorting_pool_1	5,205,492	2,450,078	0.4707	0.9867	151
2^nd^_KO_sorting_pool_2	4,927,851	2,492,036	0.5057	0.9916	151
2^nd^_KO_sorting_pool_3	5,824,112	2,719,995	0.467	0.9628	151

## Data Availability

CRISPR screening data analysis is available at https://sourceforge.net/p/mageck/wiki/Home/#usage and http://pinapl-py.ucsd.edu. Custom Python code and R code used to visualize data are available at https://github.com/Su-hyun-Kim/CHO_CRISPR_Screen.
